# Hydrotherapy after Rotator Cuff Repair Improves Short-Term Functional Results Compared with Land-Based Rehabilitation When the Immobilization Period Is Longer

**DOI:** 10.3390/jcm13040954

**Published:** 2024-02-07

**Authors:** Alexandre Lädermann, Alec Cikes, Jeanni Zbinden, Tiago Martinho, Anthony Pernoud, Hugo Bothorel

**Affiliations:** 1 Division of Orthopaedics and Trauma Surgery, La Tour Hospital, 1217 Meyrin, Switzerland; 2 Faculty of Medicine, University of Geneva, 1205 Geneva, Switzerland; 3 Division of Orthopaedics and Trauma Surgery, Department of Surgery, Geneva University Hospitals, 1205 Geneva, Switzerland; 4 Division of Orthopaedics and Trauma Surgery, Genolier Clinic, 1272 Genolier, Switzerland; 5 Synergy Medical Centre, Medbase Group, 1007 Lausanne, Switzerland; 6 Research Department, La Tour Hospital, 1217 Meyrin, Switzerland

**Keywords:** arthroscopic rotator cuff repair, immobilization duration, rehabilitation, hydrotherapy, land-based therapy, constant score

## Abstract

**Background:** The evidence of hydrotherapy after rotator cuff repair (RCR) is limited as most studies either used it as an adjuvant to standard land-based therapy, or have different initiation timing. This study aimed to compare hydrotherapy and land-based therapy with varying immobilization time. **Methods:** Patients who underwent RCR with a 10-days or 1-month immobilization duration (early or late rehabilitation) were prospectively randomized. **Results:** Constant scores significantly differed at three months only, with the best score exhibited by the late hydrotherapy group (70.3 ± 8.2) followed by late land-based (61.0 ± 5.7), early hydrotherapy (55.4 ± 12.8) and early land-based (54.6 ± 13.3) groups (*p* < 0.001). There was a significant interaction between rehabilitation type and immobilization duration (*p* = 0.004). The effect of hydrotherapy compared to land-based therapy was large at three months when initiated lately only (Cohen’s d, 1.3; 95%CI, 0.9–1.7). However, the relative risk (RR) of postoperative frozen shoulder or retear occurrence for late hydrotherapy was higher compared to early hydrotherapy (RR, 3.9; 95%CI, 0.5–30.0). **Conclusions:** Hydrotherapy was more efficient compared to land-based therapy at three months only and if initiated lately. Even though initiating hydrotherapy later brought greater constant scores at three months, it might increase the risk of frozen shoulders or retear compared to early hydrotherapy.

## 1. Introduction

Shoulder pain constitutes a highly prevalent complaint, with estimates suggesting s lifetime prevalence as high as 67% [1]. Among the numerous causes of this discomfort, rotator cuff tears (RCT) stand out, accounting for approximately one-third of reported shoulder complaints [2]. This particular pathology is among the most frequently encountered musculo-tendinous injuries seen and treated by orthopedic surgery. RCTs may emerge either due to the degeneration of tendons comprising the rotator cuff or as a consequence of trauma [3]. In degenerative rotator cuff diseases, several risk factors have been identified, with age playing a significant role in its development [4]. Consequently, this condition is notably prevalent among adults age over 50 years old and within the elderly population, with an anticipated increase in prevalence as the population continues to age [5,6,7,8,9]. The essential role of the rotator cuff in shoulder function renders addressing this pathology critical. RCTs contribute to shoulder pain, increased stiffness, and decreased strength, considerably hindering individuals in performing daily activities, even as basic as combing hair [10,11]. Moreover, this condition incurs considerable societal and economic burdens due to productivity losses and functional decline [12].

Conservative treatment has been primarily indicated for degenerative RCT, demonstrating satisfactory outcomes, particularly in addressing rotator cuff-related shoulder pain [13] or improving active forward range of motion [14]. A delayed surgical intervention, however, can increase the risk of anatomical deterioration including muscle atrophy, fatty infiltration and an increase in tear size [15]. Consequently, surgical intervention for RCT has been increasingly performed [5], either as first-line treatment or following unsuccessful conservative approaches, with improved long-term outcomes [16]. Surgical procedures encompass open interventions, mini-open approaches or arthroscopy techniques, each bearing distinct advantages and disadvantages. Although historically, open procedures prevailed [17], technological and surgical advancements led to the adoption of arthroscopic methods. Arthroscopy has become the gold standard for rotator cuff repair (RCR) as it is a minimally invasive approach reducing complications, pain, and stiffness compared to open procedure [18]. While arthroscopic RCR grants satisfactory outcomes for most patients [19], stiffness remains a common post-operative complication contributing to functional disability, pain and frustration [20]. This emphasizes the importance of post-operative rehabilitation since it helps, when supervised by physiotherapists, at reducing the occurrence of such complications and alleviate patient symptoms [21].

There are numerous rehabilitation modalities after arthroscopic RCR. The main modality that can be considered is the timing of initial mobilizations post-surgery, which can be early, delayed, or strict (no mobilization). Despite the importance of post-operative rehabilitation, there is a lack of high-quality evidence-based studies to guide clinicians, and no consensus regarding the most appropriate protocol [22,23,24]. Delayed mobilization might minimize strain at the repair site as the tendon begins to heal, potentially leading to improved healing rates [25]. Delayed range of motion, however, could maximize tendon adhesions and stiffness. On the other hand, early rehabilitation helps prevent joint stiffness, facilitating a quicker return to functionality and daily activities [23,26,27,28]. However, long-term outcomes might remain comparable to those obtained after a delayed rehabilitation, which advocates for a longer immobilization time [27,28]. Furthermore, early rehabilitation needs to be performed progressively and cautiously as it could entail a higher risk of re-rupture due to excessive load with regard to the tendon’s healing state [29]. Therefore, hydrotherapy has been introduced during early rehabilitation to diminish joint stress, aiding in shoulder mobilization for patients experiencing pain, anxiety, or dysfunctional muscular activation [30].

Hydrotherapy reduces strain, allowing patients to engage in active range of motion. This activity is essential for tissue healing from a physiological standpoint [31]. Additionally, it holds neurophysiological importance as it enhances proprioception and replicates physiological activation patterns without compromising tendon repair [30]. Despite these advantages, the evidence of hydrotherapy benefits after RCR remains limited. Most of the studies either employed it as an adjuvant therapy to standard land-based rehabilitation [30] or restricted its application to selected patients only [32,33]. Recently, two randomised clinical trials have reported contradictory findings concerning the effects of hydrotherapy compared to land-based therapy [21,34], though they differed in terms of tear size studied (small-medium vs. small-large) and immobilization duration before rehabilitation initiation (10 days or 1 month). The authors of the present study therefore aimed to investigate if, on a comparable group of patients, the benefits of hydrotherapy over land-based therapy depend on immobilization time after RCR. We hypothesized that hydrotherapy’s effects would be more pronounced if rehabilitation initiation occurs later.

## 2. Materials and Methods

The data utilized in this study originate from two clinical studies conducted by Dufournet et al. [34] and Cikes et al. [21]. Thus, patients who underwent primary arthroscopic RCR at La Tour Hospital or Bois-Cerf Clinic between 2012 and 2019 were eligible. Inclusion criteria were (1) small to medium sized symptomatic supraspinatus and/or infraspinatus tendon tears [35], (2) grade 1 to 2 tendon retraction according to Patte [36], (3) fatty infiltration stage ≤ 2 [37], and failure of conservative treatment during a minimum of six months in case of degererative lesions. Rotator cuff tears in this study were either traumatic or degenerative. Degenerative lesions were failure of conservative treatment, which involved standard land-based physiotherapy over a 6-month period. Additionally, cortisone injections were administered during this timeframe. A mandatory period of at least 3 months post-injection, was observed before the surgical intervention. Since the study performed by Dufournet et al. [34] included lesions of all sizes, 24 patients (26.1%) were not included in the present study due to the presence of large lesions. Exclusion criteria were (1) patients unable to follow the study protocol, (2) other types of rotator cuff lesion (bony rotator cuff (A), medial tendinous disruption (B2), tendon-to-tendon adhesion ‘Fosbury flop tear’ (B3), and musculotendinous junction lesion (C type)) [35], (3) patients with subscapularis tendon lesions, (4) associated superior labrum anterior posterior (SLAP) lesion, or (5) frozen shoulder [38]. In both studies, patients were randomized between the rehabilitation protocols and provided their written informed consent. Furthermore, ethical approval was granted by the local ethics committee for both (CER–VD-481/15, 13 January 2016; CCER–2016-02242, 27 July 2017), and the studies registered at ClinicalTrials.gov (NCT05106842) and our National Clinical Trials Portal (SNCTP No. 000002244).

### 2.1. Pre- and Post-Operative Clinical Assessment

Data were collected through independent assessors at baseline before the surgical intervention and at 3, 6, and 24 months post-operatively. Patients characteristics included age, sex, and dominant side. Functional status was assessed by the Constant score, a validated questionnaire ranging from a score of 0 (indicating the worst functional status) to 100 (indicating the best functional status) [39].

### 2.2. Surgical Procedure

The surgery was performed under general anesthesia and with ultrasound (US)-guided interscalene brachial plexus block with the patients placed in a beach-chair position. Adjuvant acromioplasty was performed only in patients who had radiographic signs of dynamic impingement [40], and resection of the distal part of the clavicle was performed when pain was elicited by palpation of the acromioclavicular joint. Biceps tenodesis or tenotomy was performed when the posterior wall of the bicipital groove was damaged. All repairs were carried out using two anchors, of which one was implanted at the bone–cartilage junction, and one was implanted at the lateral part of the greater tuberosity [41]. At the end of the intervention, all repairs were complete and “watertight”, with adequate restoration of the tendons to their footprints. Post-operative care included regular wound dressing twice per week with removal of skin closure sutures 10 days after surgery.

### 2.3. Rehabilitation Protocol

Patients had to wear a sling for four weeks, ensuring the positioning of the shoulder in an internally rotated stance. During the immobilization phase, patients were advised to engage in gentle self-passive motion exercises. The immobilization duration varied between two groups: patients operated and rehabilitated at La Tour hospital had a 10-day immobilization period (Early rehabilitation group), whereas patients operated and rehabilitated at Bois-Cerf Clinic underwent a 30-day immobilization (Late rehabilitation group). The early rehabilitation group started supervised physiotherapy at 10 days, after skin closure removal. The exercises consisted in progressive passive motion for three weeks, followed by active motion until the third postoperative month [42]. At three months, patients then began strengthening exercises. The Late rehabilitation group started supervised physiotherapy at one month post-operatively with progressive passive and active motion for two weeks, two to three times a week, before proceeding to strengthening exercises. In both the Early and Late groups, patients were allocated to receive either standard land-based therapy or hydrotherapy. Consequently, our final study cohort comprised four distinct groups: (1) Early rehabilitation with Hydrotherapy (Early–Hydrotherapy), (2) Late rehabilitation with Hydrotherapy (Late–Hydrotherapy), (3) Early rehabilitation with Land-based therapy (Early–Land-based), (4) Late rehabilitation with Land-based therapy (Late–Land-based). Hydrotherapy sessions were performed in a swimming pool with a depth ranging between 125 and 140 cm depth. Patients were instructed to kneel or sit to submerge their shoulders during exercises, performed in water heated to a temperature ranging between 28 to 34 °C.

### 2.4. Statistical Analyses

The sample size was determined *a priori* for both studies. In Dufournet et al. study [34], it was calculated in order to ensure the detection of a minimal clinically important difference (MCID) of 20° in active forward flexion between patients undergoing aquatic therapy and standard land-based therapy. The sample size in Cikes et al. study [21] was performed to detect a minimal clinically important difference in Constant score, corresponding to a 10.4 points change [43]. In this study, the sample size was calculated to detect at least a medium effect (f = 0.253, partial eta square = 0.06) of a physiotherapy type (aquatic vs. land-based) in postoperative Constant scores while considering the differences in therapy onset times (early vs. late). Parameters for the sample size calculation were estimated according to a ‘worst-case scenario’ approach, with low correlation among repeated measures (r = 0.2) and nonsphericity correction (ε = 0.5). To achieve a power of 0.8 in those circumstances, a minimum total sample size of 96 patients was required (24 per group).

Descriptive statistical methods were used to summarize the data. Continuous variables were reported as the mean along with the standard deviation (mean ± SD), additionally displaying the range from the minimum to the maximum values (min-max). Categorical data were reported as counts (n) and proportions. The normality of the distributions for continuous variable was assessed using the Shapiro–Wilk test and the normality of the residuals was visually assessed on a Q–Q plot. Two-way mixed ANOVA tests were conducted at each follow-up point to evaluate the effect of rehabilitation type (hydro- vs. land-based therapy) and the commencement timing of rehabilitation (Early vs. Late) on post-operative Constant scores. Effect sizes calculated with this ANOVA analysis were expressed in generalized eta squared (η^2^_G_) and interpreted as follows: small (0.01 to 0.05), medium (0.06 to 0.13) and large (≥0.14). Post-hoc analyses comparing groups of patients at each time point were conducted using Wilcoxon rank sum tests or unpaired Student t-tests. Analyses comparing patient data at different follow-up time points were performed with Wilcoxon signed rank tests or paired Student t-tests. Tests were adjusted for multiple comparisons using the Bonferroni correction. Categorical variables were compared using Chi-squared tests or Fischer tests. To evaluate and compare the effect of hydrotherapy versus land-based therapy for the two different immobilization durations at different follow-ups, Cohen effect sizes were computed and interpreted as follows: negligeable (0.00 to 0.19), small (0.20 to 0.49), medium (0.50 to 0.79) and large (≥0.80). The analyses were performed using R (version 4.1.3, R Foundation for Statistical Computing, Vienna, Austria), following the intention to treat analysis method, and with *p*-values less than 0.05 considered as significant.

## 3. Results

**Figure 1:**
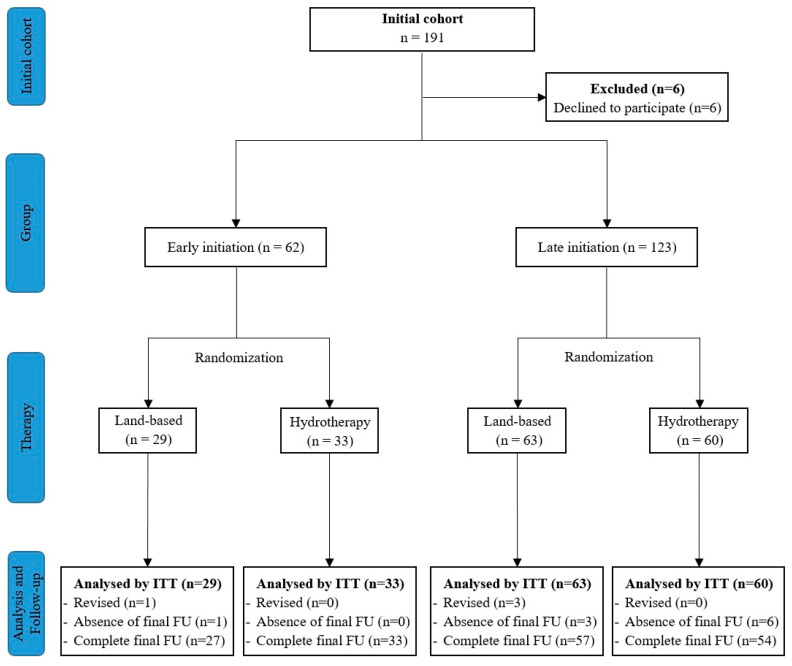
Flow diagram of patients’ selection.

A total of 191 patients were eligible and six patients declined to participate to the study (3.1%). The study enrolled a cohort of 185 patients, among whom 92 patients were allocated to land-based therapy, comprising 29 (16%) patients who commenced physiotherapy early, and 63 (34%) who initiated it at a later phase. Conversely, 93 patients underwent hydrotherapy, with 33 (18%) in the early rehabilitation group, and 60 (32%) in the late rehabilitation group (Figure 1).

**Figure 2:**
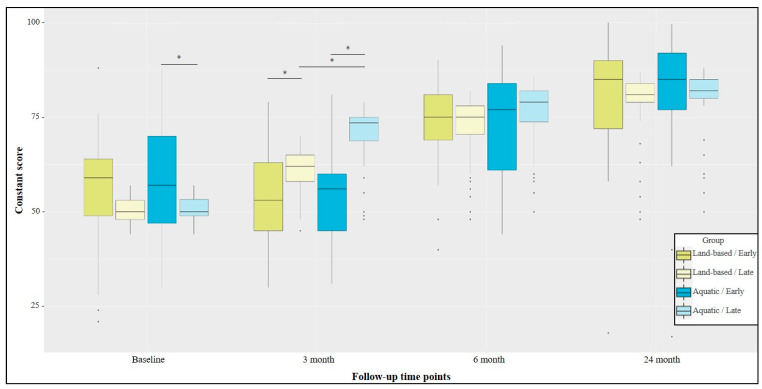
Pre-operative and post-operative Constant score depending on type of rehabilitation (Land-based therapy vs. Hydrotherapy) and the physiotherapy beginning (Early vs. Late). Black dots indicate outliers. Black stars indicate statistically significant difference between the groups at a given follow-up.

There was no statistical difference among the four groups concerning patient age (*p* = 0.121), dominancy of the affected side (*p* = 0.114) or gender distribution (*p* = 0.992) (Table 1). However, it is worth mentioning that patients allocated to the early hydrotherapy group had a slightly greater pre-operative Constant score (58.0 ± 16.7) compared with those in the late hydrotherapy group (50.6 ± 3.2) (*p* = 0.009) (Figure 2).

### Post-Operative Outcomes

Patients who initiated physiotherapy early had no improvements at three months from baseline scores, regardless of rehabilitation protocol. Patients who started physiotherapy lately, on the other hand, had an improvement at three months both for the land-based rehabilitation (mean improvement of 10.6 points, *p* < 0.001) and the Hydrotherapy (mean improvement of 19.7 points, *p* < 0.001). At three months post-operatively, patients who initiated land-based physiotherapy later showed a significantly higher Constant score than those who initiated it early (mean difference of 6.4 points, *p* = 0.042), though this difference was not clinically relevant. At the same time-point, patients who initiated lately hydrotherapy had a statistically significant higher Constant score than those who initiated it early (mean difference of 14.9 points, *p* < 0.001), with this difference being clinically relevant. From three to six post-operative months, all groups statistically improved, exceeding a difference that seems to be clinically relevant, except for the Late–Hydrotherapy group. No statistically significant difference was observed neither between the land-based therapy and aquatic therapy groups when physiotherapy was initiated early (mean difference of 1.1 point, *p* = 1.000) or at a later phase (mean difference of 3.3 points, *p* = 0.478). At 6 months, all groups plateaued and no clinically relevant improvement was observed at 24 months. Only the Late-Land-based group had a statistical improvement (mean improvement of 6.6 points, *p* = 0.003). No differences were observed at 24 postoperative months, neither between patients who initiated early and lately land-based therapy (mean difference 1.0 point, *p* = 1.000), nor between patients who initiated early and lately aquatic therapy (mean difference of 0.4 point, *p* = 1.000). Likewise, initiating physiotherapy early after surgery was not statistically superior at 24 months in the land-based group (mean difference of 0.0 point, *p* = 1.000) and hydrotherapy group (1.5 point, *p* = 1.000) (Table 2).

The two-way ANOVA revealed an effect of the rehabilitation type (*p* = 0.001), and the immobilization duration (*p* < 0.001), with an interaction between those two factors (*p* = 0.004) at a three-month follow-up solely (Table 3).

**Figure 3:**
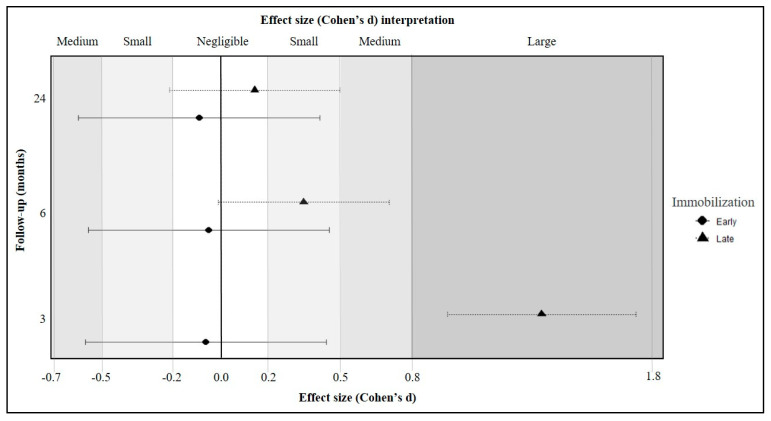
Effect size of early and late hydrotherapy (compared to land-based rehabilitation) at each follow-up time point.

Cohen effect sizes showed that, at a three-month follow-up, hydrotherapy had a large effect compared to land-based therapy when initiated later only (Cohen’s d, 1.34; 95%CI, 0.95–1.73). A tendency was also observed at 6 months post-operatively in favor of the late hydrotherapy protocol (Cohen’s d, 0.35; 95%CI, −0.01–0.70) (Figure 3).

The rate of complications was higher for patients who initiated their rehabilitation lately for both the land-based and aquatic rehabilitation groups. Patients who initiated physiotherapy lately had more revisions (2.4% vs. 1.6%) and more complications (19.5% vs. 6.5%) for those allocated to the hydrotherapy (*p* = 0.033), and we observed a tendency also for those who underwent conventional land-based therapy (*p* = 0.071).

## 4. Discussion

Hydrotherapy is an interesting modality in rehabilitation due to its capacity to facilitate shoulder mobilization while exerting lesser strain on muscles and tendons. However, evidence concerning the effects of hydrotherapy remains relatively sparse. Recent investigations by Cikes et al. [21] and Dufournet et al. [34] explored this thematic, albeit yielding contradictory outcomes. Nevertheless, there was an important methodological difference between the two studies, as the post-operative immobilization duration differed greatly, with patients included in the Dufournet et al. study having a 10-day immobilization period, while patients included in the Cikes et al. study had a 30-day immobilization period. The objective of this study was therefore to assess the potential interaction between rehabilitation type (Land-based therapy vs. Hydrotherapy) and the duration of immobilization (Early vs. Late). The main finding of this study was that hydrotherapy had a large effect at three months in improving the functional patient status (compared to land-based rehabilitation) only for those who were immobilized for longer period, confirming the interaction between the rehabilitation type and the immobilization duration.

Previous studies have shown that shoulder recovery can be accelerated using hydrotherapy rather than land-based therapy [21,32]. Aquatic therapy facilitates passive or active range of motion exercises with reduced strain on the musculo-tendinous structures. This reduced stress on muscles and tendons allows for earlier engagement of the affected shoulder, potentially enhancing improving the healing process without compromising long-term tendon integrity [32,44]. Despite the increasing interest in hydrotherapy, the current literature remains limited with studies based on small sample sizes [32,33], or where hydrotherapy is combined with standard therapy [32], thereby complicating the assessment of aquatic therapy’s independent effect. In this study, however, we identified that hydrotherapy had a particular efficient role for patients who were first immobilized for a longer period (Cohen’s d: 1.34, 95%CI [0.95–1.73], who may have stiffer shoulders [45]. Among the patients undergoing hydrotherapy, thaose who started therapy lately demonstrated a significantly higher score of 15 points at a three-month follow-up compared to those who started immediately after surgery (*p* < 0.001), exceeding the minimal clinically important difference. Likewise, Sekome et al. found beneficial short-term effects of hydrotherapy for patients experiencing knee stiffness [46]. Conversely, we found that hydrotherapy had a negligible short-term effect when initiated promptly, as patients may be less likely to develop stiffness due to rapid mobilization (Cohen’s d: −0.06 [−0.57–0.44]). Consistently, akin to the preceding studies, there was no effect of hydrotherapy at 6 months (*p* = 1.000) and at 24 months post-operatively (*p* = 1.000).

The disparities between early and delayed rehabilitation initiation have been largely reported for the traditional land-based therapy. The results, however, differ according to the studies, with some reporting an improved range of motion, function and pain up to six months [23], while others reported no differences [27,28,47]. Most of these differences are transient stiffness, and results are equivalent at a 1-year follow-up [23,25,27,48,49]. In our study, we found that traditional land-based therapy provided higher Constant score at three post-operative months when started later (*p* = 0.042). Nevertheless, this difference didn’t reach a clinical importance (mean improvement 6.4) [43]. As found in the aforementioned studies, this difference had vanished at later follow-ups (*p* = 1.000). The immobilization duration also had an effect on the two-year complication rate with patients who initiated physiotherapy lately having more complications (20% vs. 7%) and revisions (4% vs. 2%). Patients undergoing hydrotherapy early had less complications (3% vs. 12%) and less revisions (0% vs. 3%). Thus, rehabilitation protocol modalities should be guided by the desire to have good results quickly or to privilege the absence of complications in the longer term. Therefore, all patients should not necessarily be allocated to an aquatic-based therapy since the results are heterogeneous depending on the immobilization duration, and the burden (financial, temporal, etc.) that this therapy can add on the patient. However, caution is required in inferring the figures as the design of our study does not provide the necessary power to assess complication rates and revision rates. Further studies are needed to compare the long-term repair integrity associated with the rehabilitation modalities.

### Limitations

Related to the different modalities, neither clinicians nor patients were blinded to their rehabilitation. The surgical interventions were carried out by two distinct experienced surgeons operating in two different centers. However, to mitigate this potential bias, effect sizes regarding the impact rehabilitation type were computed by comparing groups within the same center. Moreover, only the Constant score was used whereas other PROMs would have been of interest such as the pain measured on a visual analog scale or the American Shoulder and Elbow Surgeons score. Additionally, these patient-reported scores are inherently subjective as they rely on patient responses and their initial health condition. Consequently, patients exposed to a longer period of immobilization might start physiotherapy in a relatively worse condition, potentially influencing their perception of improvement, thereby rating their progress more positively. Therefore, complementing these findings with more objective measures such as range of motions would indeed be beneficial and informative.

## 5. Conclusions

Hydrotherapy is a modality that provides superior results at a short-term follow-up in patients who initiated physiotherapy later compared to land-based therapy. At long-term follow-up, however, there was no difference in Constant score between the groups. This absence of discrepancy persisted irrespective of the type of rehabilitation employed or the duration of immobilization.

## Figures and Tables

**Table 1:** Demographic and pre-operative data.

	Land-Based Therapy (n = 92)					Hydrotherapy (n = 93)					*p*-Value LB vs. H	
	Early (n = 29)		Late (n = 63)			Early (n = 33)		Late (n = 60)				
	Mean ± SD	(Min–Max)	Mean ± SD	(Min–Max)	*p*-Value	Mean ± SD	(Min–Max)	Mean ± SD	(Min–Max)	*p*-Value	Early	Late
	n (%)		n (%)			n (%)		n (%)				
Male gender	16 (55%)		36 (57%)		1.000	19 (58%)		33 (55%)		0.983	1.000	0.954
Dominant side	20 (69%)		33 (52%)		0.205	25 (76%)		35 (58%)		0.146	0.754	0.630
Age at surgery	56.0 ± 7.5	(45.0–75.0)	56.8 ± 5.4	(47.0–67.0)	0.624	52.8 ± 9.5	(37.0–69.0)	56.2 ± 5.2	(46.0–67.0)	0.063	0.146	0.811
Score												
Constant	55.9 ± 15.9	(21.0–88.0)	50.4 ± 3.3	(44.0–57.0)	0.125	58.0 ± 16.7	(30.0–87.5)	50.6 ± 3.2	(44.0–57.0)	**0.009**	1.000	1.000

H, Hydrotherapy; LB, Land-based; Max, Maximum; Min, Minimum; n, Number of patients; SD, Standard deviation. Bold and underlined *p*-values indicate statistically significant differences.

**Table 2:** Comparison of post-operative scores between the rehabilitation type (Land-based vs. Hydrotherapy) and the beginning of therapy (Early vs. Late).

	Land-Based Therapy (n = 92)					Hydrotherapy (n = 93)					*p*-Value LB vs. H	
	Early (n = 29)		Late (n = 63)			Early (n = 33)		Late (n = 60)				
	Mean ± SD	(Min–Max)	Mean ± SD	(Min–Max)	*p*-Value	Mean ± SD	(Min–Max)	Mean ± SD	(Min–Max)	*p*-Value	Early	Late
Constant												
3 months	54.6 ± 13.3	(30.0–79.0)	61.0 ± 5.7	(45.0–70.0) *	**0.042**	55.4 ± 12.8	(31.0–81.0)	70.3 ± 8.2	(48.0–79.0) *	**<0.001**	1.000	**<0.001**
6 months	72.6 ± 12.3	(40.0–90.0) *	72.2 ± 8.6	(48.0–82.0) *	1.000	73.7 ± 13.7	(44.0–94.0) *	75.5 ± 9.5	(50.0–86.0) *	1.000	1.000	0.478
24 months	79.8 ± 16.2	(18.0–100.0)	78.8 ± 9.0	(48.0–87.0) *	1.000	79.8 ± 18.0	(17.0–100.0)	80.3 ± 8.6	(50.0–88.0)	1.000	1.000	1.000

H, Hydrotherapy; LB, Land-based; Max, Maximum; Min, Minimum; SD, Standard deviation; * indicates significant difference with previous follow-up (Wilcoxon signed rank test with Bonferroni correction). Bold and underlined *p*-values indicate statistically significant differences.

**Table 3:** Two-way mixed ANOVA (type III tests) for Constant score at the different follow-up.

Follow-Up	Effect	DFn	DFd	F	*p*-Value	Ges
3 month	Rehabilitation	1	181	11.786	**0.001**	0.061
	Immobilization	1	181	52.562	**<0.001**	0.225
	Rehabilitation × Immobilization	1	181	8.372	**0.004**	0.044
6 month	Rehabilitation	1	181	1.808	0.180	0.010
	Immobilization	1	181	0.2	0.655	0.001
	Rehabilitation × Immobilization	1	181	0.471	0.493	0.003
24 month	Rehabilitation	1	181	0.158	0.692	0.001
	Immobilization	1	181	0.017	0.895	0.000
	Rehabilitation × Immobilization	1	181	0.16	0.690	0.001

DF, Degrees of Freedom; F, F-Statistic; Ges, Generalized eta squared. Bold and underlined *p*-values indicate statistically significant associations.

## Data Availability

The data presented in this study are available from the corresponding author upon reasonable request.

## References

[1] Luime J.J., Koes B.W., Hendriksen I.J., Burdorf A., Verhagen A.P., Miedema H.S., Verhaar J.A. (2004). Prevalence and incidence of shoulder pain in the general population; a systematic review. Scand. J. Rheumatol..

[2] Bunker T. (2002). Rotator cuff disease. Curr. Orthop..

[3] Hashimoto T., Nobuhara K., Hamada T. (2003). Pathologic evidence of degeneration as a primary cause of rotator cuff tear. Clin. Orthop. Relat. Res..

[4] Codding J.L., Keener J.D. (2018). Natural History of Degenerative Rotator Cuff Tears. Curr. Rev. Musculoskelet. Med..

[5] Karjalainen T.V., Jain N.B., Heikkinen J., Johnston R.V., Page C.M., Buchbinder R. (2019). Surgery for rotator cuff tears. Cochrane Database Syst. Rev..

[6] Teunis T., Lubberts B., Reilly B.T., Ring D. (2014). A systematic review and pooled analysis of the prevalence of rotator cuff disease with increasing age. J. Shoulder Elb. Surg..

[7] Vidal C., Lira M.J., de Marinis R., Liendo R., Contreras J.J. (2021). Increasing incidence of rotator cuff surgery: A nationwide registry study in Chile. BMC Musculoskelet. Disord..

[8] Yanik E.L., Chamberlain A.M., Keener J.D. (2021). Trends in rotator cuff repair rates and comorbidity burden among commercially insured patients younger than the age of 65 years, United States 2007–2016. JSES Rev. Rep. Tech..

[9] Paloneva J., Lepola V., Aarimaa V., Joukainen A., Ylinen J., Mattila V.M. (2015). Increasing incidence of rotator cuff repairs–A nationwide registry study in Finland. BMC Musculoskelet. Disord..

[10] Littlewood C.M.S., Walters S. (2013). Epidemiology of Rotator Cuff Tendinopathy: A Systematic Review. Shoulder Elb..

[11] McCabe R.A., Nicholas S.J., Montgomery K.D., Finneran J.J., McHugh M.P. (2005). The effect of rotator cuff tear size on shoulder strength and range of motion. J. Orthop. Sports Phys. Ther..

[12] Parikh N., Martinez D.J., Winer I., Costa L., Dua D., Trueman P. (2021). Direct and indirect economic burden associated with rotator cuff tears and repairs in the US. Curr. Med. Res. Opin..

[13] Paraskevopoulos E., Plakoutsis G., Chronopoulos E., Maria P. (2023). Effectiveness of Combined Program of Manual Therapy and Exercise Vs Exercise Only in Patients With Rotator Cuff-related Shoulder Pain: A Systematic Review and Meta-analysis. Sports Health.

[14] Longo U.G., Rizzello G., Petrillo S., Loppini M., Maffulli N., Denaro V. (2019). Conservative Rehabilitation Provides Superior Clinical Results Compared to Early Aggressive Rehabilitation for Rotator Cuff Repair: A Retrospective Comparative Study. Medicina.

[15] Moosmayer S., Gartner A.V., Tariq R. (2017). The natural course of nonoperatively treated rotator cuff tears: An 8.8-year follow-up of tear anatomy and clinical outcome in 49 patients. J. Shoulder Elb. Surg..

[16] Chalmers P.N., Ross H., Granger E., Presson A.P., Zhang C., Tashjian R.Z. (2018). The Effect of Rotator Cuff Repair on Natural History: A Systematic Review of Intermediate to Long-Term Outcomes. JB JS Open Access.

[17] Colvin A.C., Egorova N., Harrison A.K., Moskowitz A., Flatow E.L. (2012). National trends in rotator cuff repair. J. Bone Joint Surg. Am..

[18] Tauro J.C. (1998). Arthroscopic rotator cuff repair: Analysis of technique and results at 2- and 3-year follow-up. Arthroscopy.

[19] Aleem A.W., Brophy R.H. (2012). Outcomes of rotator cuff surgery: What does the evidence tell us?. Clin. Sports Med..

[20] Namdari S., Green A. (2010). Range of motion limitation after rotator cuff repair. J. Shoulder Elb. Surg..

[21] Cikes A., Kadri F., van Rooij F., Ladermann A. (2023). Aquatic therapy following arthroscopic rotator cuff repair enables faster improvement of Constant score than land-based therapy or self-rehabilitation therapy. J. Exp. Orthop..

[22] Bouche P.A., Gaujac N., Descamps J., Conso C. (2022). Assessment of several postoperative protocols after rotator cuff repair: A network meta-analysis. Orthop. Traumatol. Surg. Res..

[23] Keener J.D., Galatz L.M., Stobbs-Cucchi G., Patton R., Yamaguchi K. (2014). Rehabilitation following arthroscopic rotator cuff repair: A prospective randomized trial of immobilization compared with early motion. J. Bone Joint Surg. Am..

[24] Nabergoj M., Bagheri N., Bonnevialle N., Gallinet D., Barth J., Labattut L., Metais P., Godeneche A., Garret J., Clavert P. (2021). Arthroscopic rotator cuff repair: Is healing enough?. Orthop. Traumatol. Surg. Res..

[25] Cuff D.J., Pupello D.R. (2012). Prospective randomized study of arthroscopic rotator cuff repair using an early versus delayed postoperative physical therapy protocol. J. Shoulder Elb. Surg..

[26] Gallagher B.P., Bishop M.E., Tjoumakaris F.P., Freedman K.B. (2015). Early versus delayed rehabilitation following arthroscopic rotator cuff repair: A systematic review. Phys. Sportsmed..

[27] Mazuquin B., Moffatt M., Gill P., Selfe J., Rees J., Drew S., Littlewood C. (2021). Effectiveness of early versus delayed rehabilitation following rotator cuff repair: Systematic review and meta-analyses. PLoS ONE.

[28] Sheps D.M., Silveira A., Beaupre L., Styles-Tripp F., Balyk R., Lalani A., Glasgow R., Bergman J., Bouliane M., Shoulder (2019). Early Active Motion Versus Sling Immobilization After Arthroscopic Rotator Cuff Repair: A Randomized Controlled Trial. Arthroscopy.

[29] Houck D.A., Kraeutler M.J., Schuette H.B., McCarty E.C., Bravman J.T. (2017). Early Versus Delayed Motion After Rotator Cuff Repair: A Systematic Review of Overlapping Meta-analyses. Am. J. Sports Med..

[30] Speer K.P., Cavanaugh J.T., Warren R.F., Day L., Wickiewicz T.L. (1993). A role for hydrotherapy in shoulder rehabilitation. Am. J. Sports Med..

[31] Levin S. (1993). Early Mobilization Speeds Recovery. Phys. Sportsmed..

[32] Brady B., Redfern J., MacDougal G., Williams J. (2008). The addition of aquatic therapy to rehabilitation following surgical rotator cuff repair: A feasibility study. Physiother. Res. Int..

[33] Burmaster C., Eckenrode B.J., Stiebel M. (2016). Early Incorporation of an Evidence-Based Aquatic-Assisted Approach to Arthroscopic Rotator Cuff Repair Rehabilitation: Prospective Case Study. Phys. Ther..

[34] Dufournet A., Chong X.L., Schwitzguebel A., Bernimoulin C., Carvalho M., Bothorel H., Ladermann A. (2022). Aquatic Therapy versus Standard Rehabilitation after Surgical Rotator Cuff Repair: A Randomized Prospective Study. Biology.

[35] Ladermann A., Burkhart S.S., Hoffmeyer P., Neyton L., Collin P., Yates E., Denard P.J. (2016). Classification of full-thickness rotator cuff lesions: A review. EFORT Open Rev..

[36] Patte D. (1990). Classification of rotator cuff lesions. Clin. Orthop. Relat. Res..

[37] Goutallier D., Postel J.M., Bernageau J., Lavau L., Voisin M.C. (1994). Fatty muscle degeneration in cuff ruptures. Pre- and postoperative evaluation by CT scan. Clin. Orthop. Relat. Res..

[38] Abrassart S., Kolo F., Piotton S., Chih-Hao Chiu J., Stirling P., Hoffmeyer P., Ladermann A. (2020). ‘Frozen shoulder’ is ill-defined. How can it be described better?. EFORT Open Rev..

[39] Constant C.R., Murley A.H. (1987). A clinical method of functional assessment of the shoulder. Clin. Orthop. Relat. Res..

[40] Ladermann A., Chague S., Preissmann D., Kolo F.C., Zbinden O., Kevelham B., Bothorel H., Charbonnier C. (2020). Acromioplasty during repair of rotator cuff tears removes only half of the impinging acromial bone. JSES Int..

[41] Collin P., McCoubrey G., Ladermann A. (2016). Posterosuperior rotator cuff repair by an independent double-row technique. Technical note and radiological and clinical results. Orthop. Traumatol. Surg. Res..

[42] Barth J., Andrieu K., Fotiadis E., Hannink G., Barthelemy R., Saffarini M. (2017). Critical period and risk factors for retear following arthroscopic repair of the rotator cuff. Knee Surg. Sports Traumatol. Arthrosc..

[43] Kukkonen J., Kauko T., Vahlberg T., Joukainen A., Aarimaa V. (2013). Investigating minimal clinically important difference for Constant score in patients undergoing rotator cuff surgery. J. Shoulder Elb. Surg..

[44] Killian M.L., Cavinatto L., Galatz L.M., Thomopoulos S. (2012). The role of mechanobiology in tendon healing. J. Shoulder Elb. Surg..

[45] Sarver J.J., Peltz C.D., Dourte L., Reddy S., Williams G.R., Soslowsky L.J. (2008). After rotator cuff repair, stiffness--but not the loss in range of motion--increased transiently for immobilized shoulders in a rat model. J. Shoulder Elb. Surg..

[46] Sekome K., Maddocks S. (2019). The short-term effects of hydrotherapy on pain and self-perceived functional status in individuals living with osteoarthritis of the knee joint. S. Afr. J. Physiother..

[47] Kim Y.S., Chung S.W., Kim J.Y., Ok J.H., Park I., Oh J.H. (2012). Is early passive motion exercise necessary after arthroscopic rotator cuff repair?. Am. J. Sports Med..

[48] Thomson S., Jukes C., Lewis J. (2016). Rehabilitation following surgical repair of the rotator cuff: A systematic review. Physiotherapy.

[49] Yi A., Villacis D., Yalamanchili R., Hatch G.F. (2015). A Comparison of Rehabilitation Methods After Arthroscopic Rotator Cuff Repair: A Systematic Review. Sports Health.

